# Clinicomycological Study of the Spectrum of Pulmonary Aspergillosis at a Tertiary Care Hospital in Central India

**DOI:** 10.7759/cureus.56147

**Published:** 2024-03-14

**Authors:** Akshay Krishna, Archana Keche, Ranganath TG, Padma Das

**Affiliations:** 1 Neuromicrobiology, National Institute of Mental Health and Neurosciences, Bengaluru, IND; 2 Microbiology, All India Institute of Medical Sciences, Raipur, Raipur, IND; 3 Pulmonary Medicine, All India Institute of Medical Sciences, Raipur, Raipur, IND

**Keywords:** allergic bronchopulmonary aspergillosis, aspergillus fumigatus-specific ige elisa, anti-aspergillus igg antibody elisa, galactomannan antigen elisa, endobronchial aspergilloma, chronic necrotizing pulmonary aspergillosis, invasive aspergillosis, pulmonary aspergillosis

## Abstract

Knowing the spectrum, prevalence, and modes of diagnosis of pulmonary aspergillosis (PA) will be beneficial to clinicians for its early diagnosis and management. This study aims to estimate the prevalence, spectrum, and role of serological tests and radiological findings in the diagnosis of PA. A total of 150 patients were suspected of having PA after obtaining relevant clinical history and radiological imaging. The patients were grouped into each spectrum of PA as invasive PA (IPA), chronic necrotizing PA (CNPA), aspergilloma, allergic bronchopulmonary aspergillosis (ABPA) based on predisposing factors, clinical and radiological findings, and the guidelines of the European Organization for Research and Treatment of Cancer/Invasive Fungal Infections Cooperative Group and the National Institute of Allergy and Infectious Diseases Mycoses Study Group (EORTC/MSG). Samples (bronchoalveolar lavage (BAL), sputum, blood) were collected from these patients and processed in a microbiology lab. BAL and sputum were subjected to microscopy by potassium hydroxide mount, calcofluor white mount, and culture. The serum was separated from blood by centrifugation and subjected to specific serological tests based on the spectrum of PA that the patient was suspected to have. For IPA, serum and BAL galactomannan antigen enzyme-linked immunosorbent assay (ELISA) was performed. For CNPA and aspergilloma, the anti-*Aspergillus *IgG antibody ELISA was performed. For ABPA, the tests performed were total immunoglobulin E (IgE) ELISA, *Aspergillus fumigatus*-specific IgE ELISA, and anti-*Aspergillus *immunoglobulin G (IgG) antibody ELISA. After compiling the clinical, radiological, culture, and serological findings, patients were diagnosed to have a particular spectrum of PA. The prevalence of IPA was 1.4%, CNPA was 4%, ABPA was 3.2%, and aspergilloma was 2.9%. CNPA was the predominant spectrum of PA in our study. Culture positivity for *Aspergillus *species was seen the highest in aspergilloma patients, followed by IPA, ABPA, and CNPA patients. *A. fumigatus* was the most common causative agent of PA, except for IPA for which *Aspergillus flavus* was the most common causative. *Aspergillus niger* and *Aspergillus terreus* were less the frequent causes of PA. A combination of radiological, microbiological, and serological tests along with clinical correlation is needed to confirm the diagnosis of PA.

## Introduction

The term “aspergillosis” refers to illness due to allergy, airway or lung invasion, cutaneous infection, or extra-pulmonary dissemination caused by species of *Aspergillus* [1]. *Aspergillus *is an opportunistic pathogen and mostly infects patients with lowered immunity due to neutropenia and/or treatment with high-dose corticosteroids or immunomodulatory drugs [2]. The primary route of acquiring infection is inhalation of the fungal spores [3]. The diseases caused by *Aspergillus *range from invasive infection, colonization, toxicoses, or allergy [4]. In immunocompromised patients, *Aspergillus *spreads through the bloodstream to many sites in the body, such as the brain, liver, and bones, leading to invasive pulmonary aspergillosis (IPA) [2,4]. Chronic necrotizing pulmonary aspergillosis (CNPA) may develop in less immunocompromised patients as a progressive necrotizing cavitary process or as an aspergilloma in a preexisting lung cavity [2,4,5]. Allergic bronchopulmonary aspergillosis (ABPA) is particularly seen in asthma and cystic fibrosis patients [2,4]. In immunocompromised patients, IPA is the most common [2,4]. CNPA is common in patients with chronic lung disease and/or mildly compromised immune systems. Patients suffering from cavitary lung disease mainly present with aspergilloma [2,4,5]. Hypersensitivity to *Aspergillus *antigens leads to ABPA [2,4]. In recent decades, fungal infections have become important causes of respiratory tract infections [6,7]. The increase in frequency is especially because of intensive cytotoxic therapy and greater use of broad-spectrum antibiotics, corticosteroids, and immunosuppressants [2]. Preexisting lung diseases act as an important predisposing factor for pulmonary aspergillosis (PA) [2,8,9]. Despite significant progress in the management of PA, the infection continues to cause morbidity and mortality mainly because of intrinsic or acquired antifungal resistance, organ dysfunction preventing the use of some agents, and the deleterious effect of severe unregulated inflammation [10,11]. This cross-sectional and observational study was planned to determine the prevalence and spectrum of PA, the predominant species of *Aspergillus *causing PA, and the role of serological tests and radiological findings in the diagnosis of PA. The primary objectives include the isolation and identification of *Aspergillus *species from sputum/bronchoalveolar lavage (BAL) of clinically suspected cases of PA and the detection of antigen and anti-*Aspergillus* antibodies in the sera of clinically suspected cases of PA. The secondary objectives include the identification of predominant species of *Aspergillus *causing PA.

## Materials and methods

Patients, presenting to the Department of Pulmonary Medicine, having signs and symptoms of PA were included in this study and given the patient information sheet. Written informed consent was taken; the patients who were willing to take part in the study were included, and their results were analyzed. This study was carried out after approval of the Research Cell and Institutional Ethics Committee (IEC) of All India Institute of Medical Sciences (AIIMS), Raipur, India. For all the patients enrolled, relevant clinical history was obtained, and radiological imaging (chest X-ray and chest CT) was done. They were grouped into IPA/CNPA/aspergilloma/ABPA based on the predisposing factors and clinical and radiological findings as per criteria given by Kousha et al. [2]. Based on respective clinical suspicion, the following tests were conducted (Table 1).

**Table 1:** Tests conducted among patients with PA IPA - Invasive pulmonary aspergillosis, CNPA - Chronic necrotizing pulmonary aspergillosis, ABPA - Allergic bronchopulmonary aspergillosis, BAL - Bronchoalveolar lavage, ELISA - Enzyme-linked immunosorbent assay

Spectrum of PA	Tests conducted
IPA	Sputum/BAL microscopy, culture serum, and BAL galactomannan antigen ELISA
CNPA	Sputum/BAL microscopy, culture anti-*Aspergillus *IgG antibody ELISA
Aspergilloma	Sputum/BAL microscopy, culture anti-*Aspergillus *IgG antibody ELISA
APBA	Sputum/BAL microscopy, culture total IgE ELISA, *Aspergillus fumigatus*-specific IgE ELISA, anti-*Aspergillus *IgG antibody ELISA

Specimen collection: Sputum/BAL and blood (serum) of patients were collected in a sterile container following the standard protocol. At least two sputum and BAL samples were collected in all the patients to rule out the chances of contamination on culture. Blood was collected from the patients using standard aseptic precautions. It was processed in the Department of Microbiology.

**Figure 1:**
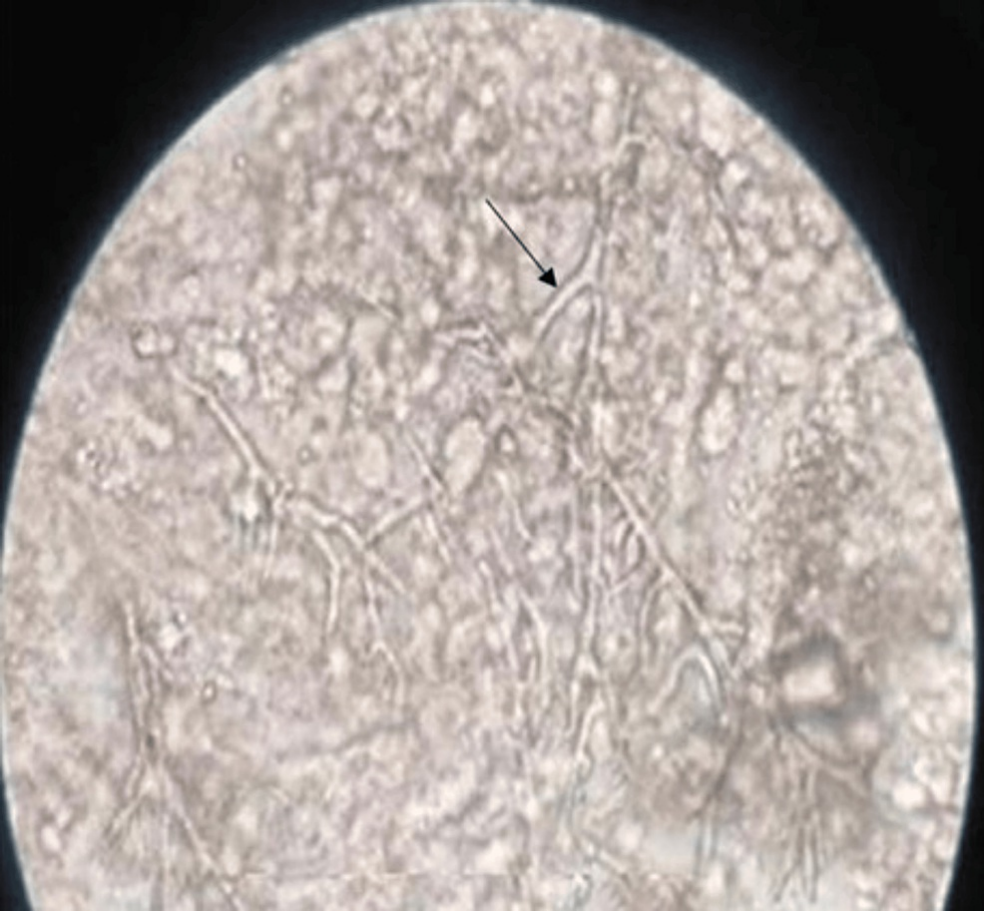
KOH mount of sputum and BAL samples of patients of PA with the arrow showing thin hyaline septate hyphae PA - Pulmonary aspergillosis; BAL - Bronchoalveolar lavage; KOH - Potassium hydroxide

Processing of samples: Sputum was subjected to N-acetyl L-cysteine (NALC) treatment, and BAL was concentrated by centrifugation (1,500-2,000 X g for five minutes) before inoculation to isolation media to enhance the detection and recovery of fungi. Microscopy of sputum and BAL was performed by means of 10% potassium hydroxide (KOH) mount preparation and 10% KOH-calcofluor white (CFW) mount preparation (Figure 1).

Culture of sputum and BAL was performed by inoculation on two sets of agars, each set having plain Sabouraud’s dextrose agar (SDA) and SDA with chloramphenicol. One set was incubated at 37°C and another at 25°C for four weeks before attributing them as negative for fungi.

Identification of the isolates was done by standard mycological laboratory procedures, which included macroscopic examination of colonies by observing growth rate, color, texture, diffusible pigment, exudates, aerial/submerged hyphae, and colony topography examination [12-14]. Microscopic examination of colonies was performed by using lactophenol cotton blue mount (LPCB) and the slide culture technique [14,15].

The serum was separated from blood by centrifugation (1,000-1,500 x g for 10 minutes). For patients suspected of having IPA, serum and BAL samples were subjected to the process of detection of galactomannan antigen by double antibody sandwich antigen enzyme-linked immunosorbent assay (ELISA) using ‘XEMA GalMAg ELISA kit’ (Xema Co., Ltd., Moscow, Russia). For patients suspected of having CNPA and aspergilloma, anti-*Aspergillus* IgG antibody ELISA was performed using IMMUNOLAB *Aspergillus fumigatus* IgG antibody ELISA test kit (Gold Standard Diagnostics, Kassel, Germany). For patients suspected of having ABPA, the tests included were the detection of total serum IgE using the total IgE EIA kit (Xema Co., Ltd), detection of *A. fumigatus*-specific IgE antibody by ELISA using a Biopanda Reagents kit and anti-*Aspergillus *IgG antibody ELISA using the IMMUNOLAB *A. fumigatus* IgG antibody ELISA test kit.

After compiling the clinical, radiological, culture, and serological findings, patients were diagnosed to have a particular spectrum of PA based on criteria given by Kousha et al. [2]. Statistical analysis was done, wherein continuous data were shown as mean and standard deviation (SD) and categorical data were shown as frequency and percentage. The chi-square test was used for categorical data analysis.

All analyses were performed using Statistical Product and Service Solutions (SPSS, version 11.0; IBM SPSS Statistics for Windows, Chicago, IL). A p-value < 0.05 was considered statistically significant.

## Results

A total of 1,294 patients presented to the Department of Pulmonary Medicine, out of which 150 patients were suspected of having PA. The mean age of PA patients was 56.9 ± 9.36 years (range: minimum 45 years and maximum 88 years) (Table 2).

**Table 2:** Age and gender distribution among patients with PA

Age group (years)	Gender		Total
	Male	Female	
18-40	-	-	-
41-60	64 (42.6%)	48 (32%)	112 (74.6%)
61-80	21 (14%)	14 (9.4%)	35 (23.4%)
>80	2 (1.4%)	1 (0.6%)	3 (2%)
Total	87 (58%)	63 (42%)	150 (100%)

Among patients with PA, the most predominant clinical history observed was asthma (27.3%), followed by chronic obstructive pulmonary disease (COPD) (12%), tuberculosis (9.3%), bronchiectasis (9.3%), and a previous history of pulmonary tuberculosis (8%). The predisposing factors among patients with PA are mentioned in Table 3.

**Table 3:** Predisposing factors among the patients with PA

Clinical history	Number of patients
Asthma	41 (27.3)
COPD	18 (12)
Tuberculosis	14 (9.3)
Bronchiectasis	14 (9.3)
Previous history of pulmonary tuberculosis	12 (8)
Diabetes Mellitus with chronic liver disease	11 (7.3)
Diabetes Mellitus	10 (6.7)
Bronchial cysts and bullae	7 (4.7)
Kidney transplantation	5 (3.3)
Chemotherapy	5 (3.3)
High-dose corticosteroid therapy	5 (3.3)
Neutropenia	4 (2.7)
Sarcoidosis	3 (2)
Alcoholism	1 (0.7)
Total	150
Numbers in parenthesis () denote percentages	

Table 4 depicts the radiological image patterns in patients with PA. All the patients with IPA showed acute respiratory distress syndrome (ARDS) pattern with pleural effusion. The most common radiological findings were consolidation with pleural thickening in CNPA patients, pulmonary infiltrates in ABPA patients, and a mass in a preexisting cavity in aspergilloma patients.

**Table 4:** Radiological image pattern in the spectrum of PA

Radiological Image pattern in the spectrum of PA			
IPA (n=19)	CNPA (n=52)	ABPA (n=41)	Aspergilloma (n=38)
Acute respiratory distress syndrome (ARDS) pattern with pleural effusion (100)	Consolidation with pleural thickening (67.2), pleural effusion (15.4), thickened and inflamed bronchi (7.7), bronchiectasis (5.7), interstitial fibrosis (4)	Pulmonary infiltrates (34.1), band-like opacities from the hilum with rounded distal margin (31.7), thickened and inflamed bronchi (22), pulmonary infiltrates with thickened and inflamed bronchi (12.2)	Mass in a preexisting cavity (52.6), mobile, intra-cavitary mass with an air crescent in the periphery (47.4)
Numbers in parenthesis () denote percentages			

Clinical diagnosis was made by the attending physician based on signs and symptoms in suspected cases of PA [2]. Cases were clinically diagnosed to have CNPA (34.6%), followed by ABPA (27.3%), aspergilloma (25.3%), and IPA (12.6%) (Table 5).

**Table 5:** Clinical diagnosis of patients with PA

Clinical diagnosis	Number of patients
Sputum production, dyspnea, pleuritic chest pain- suspected IPA	19 (12.7)
Chronic productive cough, fever, malaise, weight loss, fatigue-suspected CNPA	52 (34.6)
Episodic wheezing, chest tightness, expectoration of sputum (brownish discoloration), fever-suspected ABPA	41 (27.3)
Hemoptysis, cough, dyspnea-suspected aspergilloma	38 (25.3)
Total	150
Numbers in parenthesis () denote percentages	

Table 6 depicts the spectrum of PA among clinically suspected patients who were confirmed by diagnostic criteria based on clinical, radiological, and microbiological findings. Nineteen patients who were diagnosed with IPA were further classified as proven IPA (n = 7/19, 36.8%), probable IPA (galactomannan +, culture neg) (n = 3/19, 15.8%), and possible IPA (n = 9/19, 47.3%). A total of 52 patients were diagnosed with CNPA, 38 patients were diagnosed with aspergilloma, and 41 patients were diagnosed with ABPA according to the criteria given by Kousha et al. [2].

**Table 6:** Spectrum of pulmonary aspergillosis based on the diagnostic criteria

Spectrum of pulmonary aspergillosis	Diagnostic criteria				
	Clinical	Radiological	Microbiological (culture/microscopy)	Serological	Total (n)
IPA	19	19	7	4 (Galactomannan antigen +)	19 (12.6%), Proven – 7, Probable – 3, Possible - 9
CNPA	52	52	16	13 (IgG +)	52 (34.6%)
ABPA	41	41	15	16 (Total IgE +) 16 (specific IgE +) 23 (IgG antibody +)	41 (27.3%)
Aspergilloma	38	38	22	12 (IgG)	38 (25.3%)

**Figure 2:**
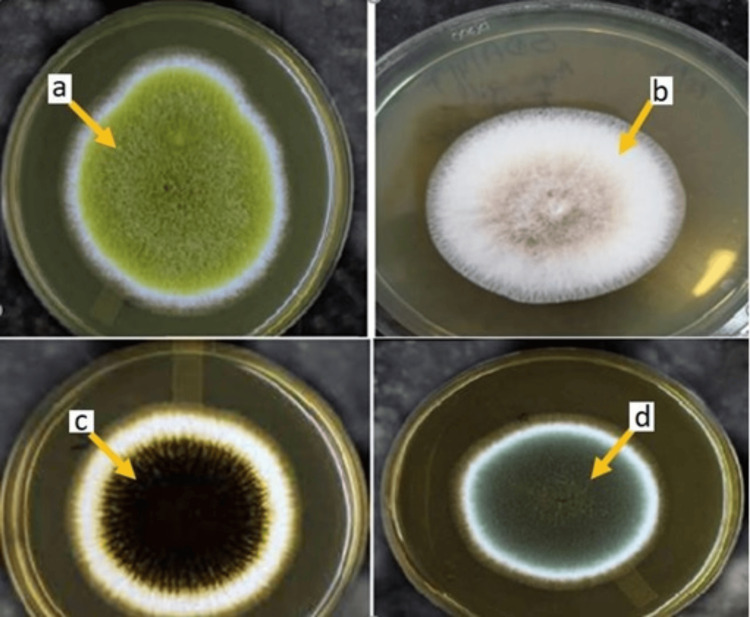
Culture growth of Aspergillus species in BAL and sputum of patients with PA a - *Aspergillus flavus*, b - *Aspergillus terreus*, c - *Aspergillus niger*, d - *Aspergillus fumigatus*

**Figure 3:**
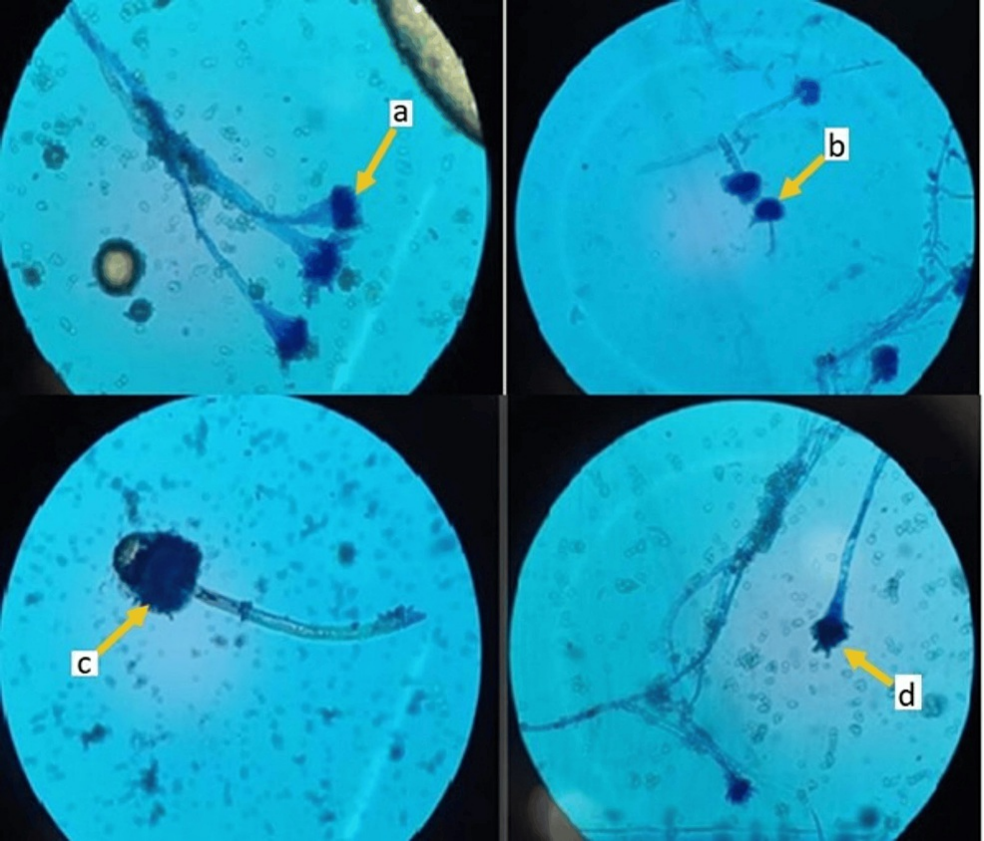
LPCB mount of Aspergillus species with arrows showing conidiophores and phialides LPCB - Lacto phenol cotton blue, a - *Aspergillus flavus*, b - *Aspergillus terreus*, c - *Aspergillus niger*, d - *Aspergillus fumigatus*

The prevalence of IPA was 1.4%, CNPA was 4%, ABPA was 3.2%, and aspergilloma was 2.9%. Various species of *Aspergillus *were isolated from BAL and sputum samples of patients with PA (Table 7, Figures 2-3). The predominant species isolated was *Aspergillus flavus* in IPA and *A. fumigatus* in CNPA, ABPA, and aspergilloma.

**Table 7:** Summary of various species of Aspergillus isolated among patients with PA

Culture findings	IPA	CNPA	ABPA	Aspergilloma	Total PA
Aspergillus fumigatus	2	6	12	9	29 (19.4%)
Aspergillus niger	1	5	1	9	16 (10.6%)
Aspergillus flavus	3	2	1	4	10 (6.6%)
Aspergillus terreus	1	3	1	0	5 (3.4%)
Total	7 (4.7%)	16 (10.6%)	15 (10%)	22 (14.7%)	60 (40%)

## Discussion

In the present study, among a total of 150 patients with PA, it was found that the middle-aged group (41-60 years) was mostly affected [16-19]. Males were more commonly affected than females [16-18]. The most predominant clinical history observed was of asthma, followed by COPD, tuberculosis, bronchiectasis, and a previous history of pulmonary tuberculosis, among many others. Immunosuppressive conditions among PA patients included corticosteroid therapy, chemotherapy, solid organ transplantation, and neutropenia, as reported in other studies [4,16-18,20-22]. This evidence points out that underlying chronic diseases are a major predisposing factor to the development of PA and patients with chronic lung diseases need to be screened for aspergillosis.

In the present study, patients with PA had a wide array of radiological findings. The ARDS pattern with pleural effusion was observed in all the patients with IPA, indicating extensive lung involvement, whereas only 20-30% of IPA patients were reported to have the ARDS pattern [17,23]. The reason for the difference in results might be due to patients reporting late to the hospital in the advanced stage of the disease. The predominant radiological finding among CNPA patients was consolidation with pleural thickening similar to findings in other studies in patients with chronic lung diseases [17,18]. Pulmonary infiltrates were mostly noticed in ABPA patients, which is consistent with the report by Agarwal et al. [24]. The majority of patients with aspergilloma had a mass in preexisting cavities similar to the reports by Smith et al. [25] and Gupta et al. [26]. This indicates that aspergilloma develops as a mass in preexisting cavities of the lung, which might have been formed due to other lung diseases, such as pulmonary tuberculosis and bronchiectasis.

The prevalence of IPA was 12.7%, CNPA was 34.6%, ABPA was 27.3%, and aspergilloma was 25.3% among a total of 150 patients with PA.

Among 19 patients with IPA, direct microscopy of BAL and sputum samples revealed fungal elements (thin hyaline septate hyphae) in 21% of patients. The culture was positive in 37% of patients, which was similar to the study by Zhou et al. [27] who reported 40% culture positivity. Culture positivity is significant in the presence of risk factors causing immunodeficiency and can only support, but not confirm, a diagnosis of PA given that *Aspergillus *is a ubiquitous pathogen [27,28]. Empirical treatment using antifungal drugs where the patients were admitted previously may be the reason for low culture positivity. Low rates of culture positivity limit the use of culture of respiratory tract secretions as a diagnostic tool. Similarly, negative cultures do not rule out a diagnosis when clinical suspicion is high and supported by radiological and serological data. *A. flavus* was the predominant species, followed by *A. fumigatus, A. niger, and A. terreus* in this study, whereas *A. fumigatus* was the predominant species isolated in earlier studies [29,30].

Galactomannan antigen test positivity was 21% in patients with IPA, which was similar to previous studies [23,27]. In contrast, galactomannan antigen positivity was higher in the study of Bretagne et al. [31] as their study population included only neutropenic patients. Decreased galactomannan antigen assay positivity may be due to antifungal therapy, which significantly lowers the assay sensitivity. The presence of *Aspergillus-*specific antibodies in these patients causes direct binding of these antibodies to the galactomannan antigen, which lowers the sensitivity of the galactomannan antigen test significantly [28]. The sensitivity and specificity of the galactomannan antigen test were found to be 14% and 75% with culture as the gold standard with no significant association between the galactomannan antigen test and culture. This may be because galactomannan antigen is released primarily by growing hyphae, and not by conidia that are colonizing the airways [21]. It is reported that difficulty in diagnosing the galactomannan antigen is due to irregular and transient excretion of antigen and formation of undetectable immune complexes [32]. Nineteen patients who were diagnosed with IPA were further classified as proven IPA (36.8%), probable IPA (15.8%), and possible IPA (47.3%) based on the European Organization for Research and Treatment of Cancer/Mycosis Study Group (EORTC/MSG) criteria [2]. Earlier studies have reported varying percentages of IPA cases [27,29,31].

CNPA is an indolent, locally invasive form of PA that often presents in patients with either preexisting lung disease or immune suppression. Among 52 patients with CNPA, microscopy showed the presence of fungal elements (thin hyaline septate hyphae) in 19.2% of patients. Culture positivity was 30.8%. *A. fumigatus* was the predominant species isolated, followed by* A. niger*, *A. terreus,* and *A. flavus*, which is similar to other studies [16,30]. These findings point out *A. fumigatus* being the most common causative agent of CNPA. *A. fumigatus*-specific IgG antibodies were positive in 25% of patients, which was similar to the study by Khurade et al. [20], but less than the result reported in the study by Yu et al. [23]. There was no significant association between *A. fumigatus*-specific IgG and culture in patients with CNPA. *A. fumigatus*-specific IgG may remain negative even in the presence of symptoms, radiology, and laboratory diagnostics suggestive of CNPA because there is a lack of appropriate antibody response to *Aspergillus *because of hypogammaglobulinemia [28]. The absence of *Aspergillus *IgG antibodies is not a definitive tool to exclude the diagnosis. The presence of anti-*Aspergillus *IgG antibodies differentiates between infected and colonized patients [33]. In the present study, *Aspergillus-*specific IgG ELISA was 25% sensitive and 66% specific with culture as a gold standard. Additionally, 75% of CNPA patients were negative for *A. fumigatus*-specific IgG, but the signs and symptoms with which they presented to the hospital correlated with CNPA infection, suggesting that the patients’ immunity was compromised and *Aspergillus *was a pathogen in them and not a colonizer. There was no significant association between *A. fumigatus*-specific IgG test and culture. In contrast, Guo et al. [34] reported a significant association between IgG and culture, and *Aspergillus*-specific IgG ELISA was 70% sensitive and 82% specific.

ABPA is an allergic disorder that happens due to hypersensitivity reactions to *Aspergillus*. In the presence of asthma and cystic fibrosis, defective clearance of fungal conidia allows the germination of conidia to hyphae, which then induces the production of proinflammatory cytokines that are responsible for the development of symptoms [4]. Among 41 patients with ABPA, microscopy showed the presence of fungal elements (thin hyaline septate hyphae) in 26.8% of patients. The culture positivity rate among ABPA patients was 36.5%. *A. fumigatus* was the predominant species isolated, followed by *A. niger, A. terreus*, and *A. flavus*. Prasad et al. [35] reported culture positivity of 30% among ABPA patients with *A. fumigatus* being the only species isolated in all the cases. Perfect et al. [30] reported that *A. fumigatus* was the most common species isolated in 92% of cases, followed by *A. flavus* in 6% of cases. This shows that *A. fumigatus* is the predominant species causing ABPA. Serum total IgE was significantly raised in 39% of patients. *A. fumigatus*-specific IgE was positive in 39% of patients. *A. fumigatus*-specific IgG was positive in 56.1% of patients. There was a significant association between the total IgE and *A. fumigatus*-specific IgE because all the patients who had significantly raised total IgE levels were also positive for *A. fumigatus*-specific IgE. The *A. fumigatus*-specific IgG positivity rate was 56.1%, which was higher compared to other studies [22,35,36]. Serum total IgE was raised in 39% of patients, which is higher than that reported by Nath et al. [37] and Sharma et al. [22] and lower than that reported by Agarwal et al. [24]. Serum *A. fumigatus*-specific IgE was positive in 39% of patients, which was similar to other studies [22,24,35,37].

Aspergilloma is a fungus ball composed of *Aspergillus *hyphae along with cellular debris and mucus and is noninvasive. Among 38 patients with aspergilloma, the KOH mount of sputum samples showed the presence of fungal elements (thin hyaline septate hyphae) in 23.7% of patients. Culture positivity was 58% among aspergilloma patients, which is higher compared to reports in other studies [26,38]. *A. fumigatus* was the predominant species isolated, followed by *A. niger* and *A. flavus*, which is similar to other studies [26,30,38]. In aspergilloma, IgG antibodies to *Aspergillus *help determine significant exposure to *Aspergillus *and thus differentiate between the transient/non-pathogenic presence of this fungus and a true infection [21]. In this study, *A. fumigatus*-specific IgG antibodies were detected in 32% of aspergilloma patients, which was similar to results in other studies [26,38].

Limitations of the study: Polymerase chain reaction (PCR) was not performed for diagnosing IPA since, at the time of commencement of our study, PCR was not included in the diagnostic criteria of IPA by EORTC/MSG. In 2020, *Aspergillus *PCR was included in consensus guidelines for defining IPA, with the requirement of two positive results providing sufficient specificity to confirm a diagnosis.

## Conclusions

The spectrum of PA among clinically suspected patients was confirmed by diagnostic criteria based on a combination of clinical, radiological, microbiological, and serological findings. CNPA was the most common spectrum of PA, followed by ABPA, aspergilloma, and IPA. *A. fumigatus* was the most common causative agent of PA, followed by *A. niger, A. flavus, *and *A. terreus*. Culture, galactomannan antigen test, and serum *Aspergillus-*specific IgG antibody could support the diagnosis of PA but had a low positivity rate. Serum total IgE and *Aspergillus-*specific IgE antibody detection were useful in the diagnosis of ABPA. A combination of radiological, microbiological, and serological tests along with clinical correlation is needed to confirm the diagnosis of PA.

## Appendices

**Table 8:** Appendix 1 - Patient information sheet

Patient information sheet	
Title of project	CLINICOMYCOLOGICAL STUDY OF THE SPECTRUM OF PULMONARY ASPERGILLOSIS AT AIIMS, RAIPUR
Purpose of the research	Aspergillosis is a common clinical entity affecting various organs, but most commonly it affects the lungs and causes pulmonary aspergillosis. It is predominant in immunocompromised patients, but few may affect immunocompetent leading to severe complications. For early diagnosis and initiation of appropriate therapy, this study is being done.
Participant’s selection: Voluntary participation	Your participation in this study is entirely voluntary. It is your choice whether to participate or not. If you choose not to participate, all the services you receive at this hospital will continue and nothing will change.
Procedure	If you consent to be a participant in this study, you will be asked to give your demographic details. The following samples are required for the study: Sputum, BAL, and Serum will be collected by the clinicians or trained staff based on clinical diagnosis.
Study duration	The total study duration for the proposed study is 18 months. But you have to participate in this only once.
Risk	This is an observational study and at least one or two samples will be required. Also, one-time interaction with the investigator is required. No follow-up visit is required for study purposes. There will be only a collection of samples and interaction with the investigator to record demographic data in Case Record Form. Hence, no/minimal risk is involved in this study.
Right to refuse or withdraw	Your participation in the proposed study is completely voluntary and you have the right to refuse or withdraw at any time during the study period. Your withdrawal from this study will not affect your routine treatment and all the services you receive at this hospital will continue and nothing will change.
Confidentiality	The information about you will be kept confidential and available to the research team only. Data generated from this study will be accessible to the research team only. The study will be presented and/or published, without disclosing your identity.
Cost to the participant	This is an observational study, and no expenses will be incurred by the participants.
Contact persons	For further information/questions, you can contact the Principal Investigator.

**Table 9:** Appendix 2 - Patient consent form

Consent form for participants	
Participant’s Name	
Age/Sex	
Date	
Address	
Title of the project	“Clinicomycological Study of the Spectrum of Pulmonary Aspergillosis at AIIMS, Raipur”
The details of the study have been provided to me in writing and explained to me in my own language. I confirm that I have understood the above study and had the opportunity to ask questions. I understand that my participation in the study is voluntary and that I am free to withdraw at any time, without giving any reason, without the medical care that will normally be provided by the hospital being affected. I agree not to restrict the use of any data or results that arise from this study provided such a use is only for scientific purpose(s). I have been given an information sheet giving details of the study. I fully consent to participate in the above study	
Signature of the participant/Thumb impression	
Date	
Signature of the witness	
Date	
Signature of the investigator	
Date	
Statement by Researcher/person taking consent	I have accurately read out the information sheet to the potential participant, and to the best of my ability made sure that the participant understands the purpose of the study. I confirm that the participant was given an opportunity to ask questions about the study, and all the questions asked by the participant have been answered correctly and to the best of my ability. I confirm that the individual has not been coerced into giving consent, and the consent has been given freely and voluntarily.
